# Evaluation of artificial neural network algorithms for predicting the effect of the urine flow rate on the power performance of microbial fuel cells

**DOI:** 10.1016/j.energy.2020.118806

**Published:** 2020-12-15

**Authors:** A. de Ramón-Fernández, M.J. Salar-García, D. Ruiz Fernández, J. Greenman, I.A. Ieropoulos

**Affiliations:** a Department of Computer Technology, University of Alicante, Alicante, E-03690, Spain; b Bristol BioEnergy Centre, Bristol Robotic Laboratory, Block T, University of the West of England, Bristol, Coldharbour Lane, Bristol, BS16 1QY, UK

**Keywords:** Artificial neural networks, Modelling, Microbial fuel cells, Urine, Flow rate, Bioenergy

## Abstract

Microbial fuel cell (MFC) power performance strongly depends on the biofilm growth, which in turn is affected by the feed flow rate. In this work, an artificial neural network (ANN) approach has been used to simulate the effect of the flow rate on the power output by ceramic MFCs fed with neat human urine. To this aim, three different second-order algorithms were used to train our network and then compared in terms of prediction accuracy and convergence time: Quasi-Newton, Levenberg-Marquardt, and Conjugate Gradient. The results showed that the three training algorithms were able to accurately simulate power production. Amongst all of them, the Levenberg-Marquardt was the one that presented the highest accuracy (R = 95%) and the fastest convergence (7.8 s). These results show that ANNs are useful and reliable tools for predicting energy harvesting from ceramic-MFCs under changeable flow rate conditions, which will facilitate the practical deployment of this technology.

## Highlights

• ANNs are reliable tools for predicting the power performance of ceramic-MFCs.
• QN, LM and CG algorithms were able to accurately simulate the power output by MFCs.
• LM algorithm showed the highest accuracy (R = 95%) and the fastest convergence (7.8s).

## 1 Introduction

Microbial Fuel Cell (MFC) technology relies on bacterial metabolism to turn the chemical energy stored in a substrate into electricity. The ability of microbes to degrade organic matter allows these systems to produce bioenergy and treat wastes simultaneously [1,2]. Respiring bacteria drive the oxidation of the substrate in the anodic compartment whereas in the cathode takes place the reduction of an oxidant, usually oxygen. Anodic and cathodic compartments are physically separated by a selective membrane, with both electrodes being connected through an external circuit which allows the electrons flow from the anode to the cathode [3]. One of the most important benefits of MFCs over other technologies is the possibility of exploiting a broad variety of substrates of waste nature, e.g. domestic and industrial wastewaters. Among them, human urine has gained much attention as a feedstock for these types of bioelectrochemical systems. Its abundance and natural properties such as high conductivity and nitrogen content, are benficial for green energy production [[4], [5], [6]].

In order to facilitate the scalability of this technology, and consequently its real implementation, much progress has been made in terms of material development, reactor design and optimisation. The search for new materials focuses on reducing the cost and improving the energy efficiency of the overall system whereas the novel MFC designs aim to facilitate the transition from the laboratory scale to its practical deployment, reducing the maintenance requirements [7]. The modification of the anode with conductive polymers has been demonstrated to be a suitable way to enhance the energy harvesting from MFCs. Wang et al. (2020) reported to reach up to 515 mW cm^−2^ by using self-supporting polyaniline-sodium alginate/carbon brush (PANI-SA/CB) hydrogel as an anode (1.38 times higher than the bare anode) [8]. The same authors improved these results by integrating self-supporting polypyrrole-carboxymethyl cellulose-titanium nitride/carbon brush hydrogel (PPy-CMC-TiN/CB). In this case, the modified bioanodes allowed to increase the power output up to 4.72 times, compared with the bare electrode [9]. Low-cost catalysts have also been synthesised as alternative materials to expensive noble metals for accelerating the oxygen reduction reaction on the cathode. For instance, recently Xin et al. (2020) elaborated Cu_2_O decorated reduced graphene oxide composite (Cu_2_O/rGO) to be used as a catalyst in MFCs. The 3D cathodes designed allowed MFCs not only to reach a higher value of power output than using a platinum-based catalyst (up to 1362 mW cm^−2^) but also improve the wastewater treatment capacity (71.5% of chemical oxygen demand removal) [10]. The combination of iron and nitrogen has also been used to synthesise alternative catalysts for the oxygen reduction reaction in MFCs. Gadja et al. (2018) reported that the performance of MFCs enhaced more than 68% when aminoantipyrine is used as a nitrogen precursor for preparing Fe–N-based catalysts, compared with those working with cathodes containing activated carbon [11]. However, when streptomycin is used as a nitrogen precursor to elaborate similar cathodes, the power output by the MFCs increased up to 74% compared with those using activated carbon as a catalyst [12].

The advancements in new materials and designs not only improve the power performance of this technology but also promote its use for practical applications such as lighting or powering electronic devices [[13], [14], [15], [16]]. However, despite the success of these field trials, most of the improvements reported so far are mainly made by running laboratory assays, which are usually cost and time demanding, and rarely represent real-world conditions. For these reasons, the use of mathematical modelling to simulate and optimise MFC performance has gained much attention in the last few years. These techniques allow us to cover multiple scenarios simultaneously under more realistic conditions. So far, mathematical models have been commonly used to model all the phenomena which take place in an MFC. However, these kinds of models usually require an in-depth knowledge of the system, which is difficult in complex systems such as MFCs [17,18]. Alternative modelling techniques based on artificial intelligence (AI) has strongly burst in a broad domain such as health, environmental sciences or biotechnology.

Artificial neural networks (ANNs) represent a very important and constantly evolving field within AI. They are mathematical models inspired by the functioning of biological neural networks. They consist of a set of simple processing units called nodes or neurons organised in layers with a high level of connection between them that allow the input signals to be transmitted through the different layers until generating output values. The following elements are in any model based on a neural network approach [19]:

• Input signals (*x*_*1,*_ *x*_*2, …*_*x*_*n*_), which may be external data or provided by other neurons.
• Output signals (*y*_*1,*_ *y*_*2, …*_*y*_*m*_), which represent the network output values.
• Weights (*w*_*1k,*_ *w*_*2k, …*_*w*_*nk*_), which represent the interaction between two neurons. The weights are modified during the training of the network in order to obtain the expected output values.
• Biases (*ϴ*_*1,*_ *ϴ*_*2,*_ *…ϴ*_*k*_), which are values associated with each node and allow the activation function to be shifted to the left or right, to better fit the data.

ANNs can be classified according to different criteria such as their topology (single or multilayer), the type of learning (supervised or unsupervised) or the type of connection between layers (feed-forward or feedback) [19].

As in other research fields, the use of ANNs to model the performance of MFCs is focusing the attention of many researchers, increasing the number of published papers in the last few years [20]. ANNs have been recently used to predict the feed substrate from 69 different microbial communities. In this work, the authors designed 6-machine learning algorithms based on 4-input variables to identify the feed substrate from genomic data. The model showed a maximum accuracy of 93 ± 6% by using an ANN trained on datasets classified at the phylum taxonomic level [21]. This methodology has also been used to predict the anodic biofilm communities and MFC performance caused by changes in the feedstock composition with a total of 33-tests performed. In this case, the authors were able to predict the power density output by the system with a minimum error of 5.76 ± 3.16% when the data were taxonomically classified at the family level [22].

The stabilisation period of the power output by MFCs has also been predicted by ANNs. This model reported that the power generation by these systems needs between 12 and 16 weeks to be stable [22]. One of the most recent work is published by Tsompanas et al. [23] and involves the study of ceramic MFCs fed with a real waste stream. In this work, ANNs are used to simulate the polarisation curves of different MFC set-ups. With a total number of 264 experiments, the authors’ model reached an accuracy of 99.66% in the data prediction.

ANNs have also been used to predict the effect of specific design parameters such as the anode angle with respect to the inlet flow direction on the electricity generated by MFCs. The experimental results obtained by Jaeel et al. [24] showed that MFCs reached the maximum power output (486 mW m^−2^) when the anode is perpendicular to the substrate flow direction at the lowest feed flow rate. In this case, a three-layer ANN model was used to predict the efficiency of the systems in terms of power production. The model revealed a good-fitting between the experimental and simulated data with a correlation coefficient of R = 99.889%. A three-layer ANN model was also used by Ismail et al. [25] in 2017 to predict the power generation of double-chamber MFCs continuously fed with domestic wastewater enriched with giant reed as a new energy source. The ANN approach presented in this work reported a good-fitting between the experimental and predicted data (R = 99.93%), which shows the potential application of AI modelling tools to optimise the performing of complex systems such as MFCs. More recently, Ali et al. [26] performed an experimental assay to optimise the MFCs set-up for maximising the electricity generation. The authors studied the effect of different factors, e.g. types of salt bridges as separators, as well as different salt concentrations, temperature or the surface area of electrodes, on the voltage generated by MFCs. A four-layer feed-forward ANN approach was employed by the authors for simulating the voltage generated by the systems. The model developed was able to predict the voltage production with a correlation between the experimental and simulated data of 99.9%.

In line with the growing interest of the scientific community in this field, in the present work the power performance of ceramic-based MFCs continuously fed with neat human urine was simulated by ANNs. Terracotta clay membranes with different porosities and bulk resistances, as well as the urine flow rate were used as input variables. 16-different feed flow rates were applied on the MFCs including 6-different ceramic membranes in triplicate, with a total number of 288 tests run. In order to obtain the best model to predict the MFC power performance, several topologies of ANN designs were studied and three training algorithms were compared according to their convergence velocities in training and performances in testing.

## 2 Materials and methods

### 2.1 Microbial fuel cell set-up

**Fig. 1:**
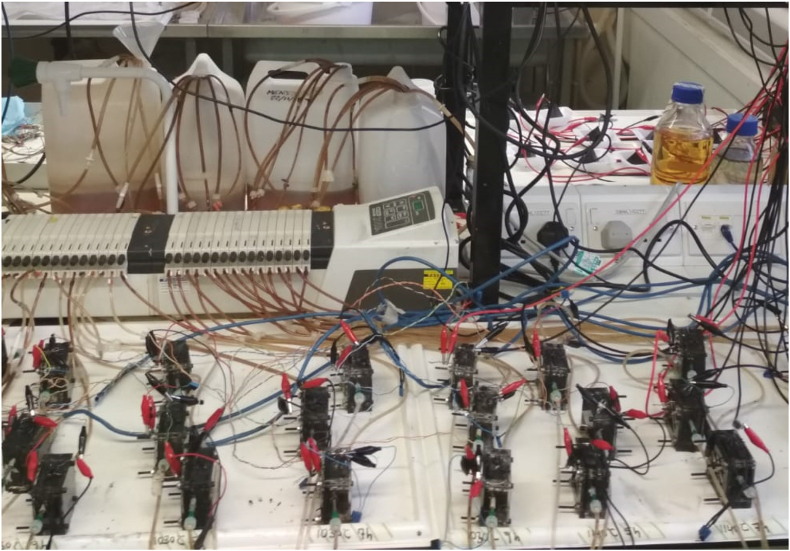
Image of the experimental set-up involving 6-MFC groups run in triplicate and fed at 16 different feed flows with a total number of 288 assays.

Cuboid single-chamber MFCs made of acrylic were used to perform the experiments. The cathodes consisted of a paste of activated carbon and polytetrafluoroethylene (PTFE) as a binder, pressed over a piece of stainless steel (12.25 cm^2^). Regarding the anodes, a piece of carbon veil (20 g m^−2^, PRF composites, Dorset, UK) coated with activated carbon were placed in the anodic compartment and connected externally to the cathode through a chromium-nickel cable [27]. As a membrane, flat square pieces of terracotta clay (12.25 cm^2^) were cut and kilned by using different procedures, which involve different combinations of temperature and ramp time. The different kilning methods allowed finely tuning membrane properties such as porosity, bulk resistance or pore size (see Table 1). The final thickness of the membranes was 3 mm. In this work, 6-different ceramic membranes were elaborated and assessed as MFC separators in triplicate, as can be seen in Fig. 1.

**Table 1:** Depiction of the 96-conditions set run in triplicate to perform the 288 essays.

Group Name	Bulk resistance (Ω)	Porosity (%)	Feed flow rate (mL.min^−1^)
1	64	26.6	0.06 0.19 0.27 0.38 0.49 0.58 0.68 0.72 0.92 0.98 1.18 1.33 1.54 1.75 1.95 3.88
2	70.6	26.1	
3	92	27.7	
4	114.2	25.8	
5	124	27.1	
6	497.2	16.8

### 2.2 Microbial Fuel Cell inoculation

The air-breathing single-chamber MFCs were inoculated with a solution containing urine and sludge (1:1 v/v) in batch mode, which was replenished daily during 4 days. Then, the systems were continuously fed with neat human urine at a flow rate of 0.06 mL min^−1^ and the external loading gradually adjusted. After 3 months working under these conditions and once the systems were completely stable and the biofilm well developed through the anode, the feed flow was slowly increased up to 3.88 mL min^−1^ at a fixed external loading of 500 Ω. The effect of 16-different feed flows on the power output by the MFCs was experimentally assessed (see Table 1) with a total number of 288 tests run. A multichannel Agilent recorder data logger (LXI 34972A data acquisition/Switch unit) was used to continuously monitor the MFC voltage.

### 2.3 Porosity analysis

The total porosity of the terracotta clay membranes was analysed by mercury intrusion porosimetry (Poremaster-60 GT, Quantachrome Instrument, United Kingdom). This device includes dual high-pressure transducers, which allow it to improve the accuracy of the results, as well as two built-in automated low-pressure ports. This system is able to make intrusion/extrusion analysis from vacuum to 60.00 psi.

### 2.4 Impedance spectroscopy (IES)

The IES technique was used to determine the bulk resistance (R_b_) of the terracotta clay membranes by using μAutoLab III with a frequency response analyser FRA2. The measurements were performed in the frequency range of 100 kHz to 10 mHz, at AC amplitude of 10 mV, in a two-electrode configuration. The value of R_b_ was determined by the intersection of the semicircle with the Z’ axis in the Nyquist plot [28,29].

### 2.5 Artificial neural network. Multilayer perceptron

**Fig. 2:**
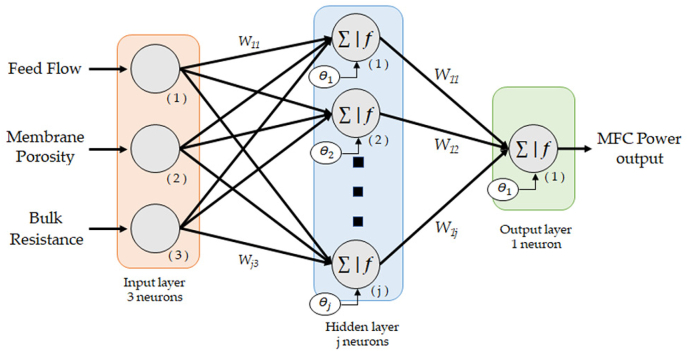
MLP architecture based on the input and output variables of our system.

A standard type of ANN, namely multilayer perceptron (MLP), was selected for the prediction of the power performance by MFCs from three input variables: feed flow, bulk resistance, and porosity of the membrane (see Fig. 2). The MLP is one of the most used neural network models since it is capable of acting as a universal approximating function. MLP emerges as an evolution of the single-layer perceptron developed by Rosenblatt in 1958 [30], which allowed solving only linearly separable problems. In 1969, Minsky and Papert [31] suggested the combination of several single-layer perceptrons to overcome this limitation and solve some non-linear challenges, thus creating a multilayer neural network of forwarding propagation. The neurons in the input layer are responsible for receiving signals from outside and propagating these signals to the output layer through the hidden layers. The neurons of this layer perform non-linear processing of the received signals. Our MLP will consist of a single hidden layer since usually most of the existing problems can be solved with this configuration, which also reduces the computation time.

The propagation rule determines the potential resulting from the interaction of neuron *i* with the *n* neighbouring neurons. One of the most common and simple rules is the weighted sum of the inputs with their corresponding weights and biases:

(1) $$net_{i}=\sum_{j=1}^{n}w_{ij}x_{j}+\theta_{i}$$

The activation function f is responsible for sending the value obtained in the propagation rule and defining the output of the layer. Here, the tangent sigmoid function was used for the hidden and output layer. It is one of the most used activation functions and is defined in the interval [-1, 1] by the following equation:

(2) $$f_{tansig}\left(x\right)=\frac{2}{1+e^{-2x}}-1$$

From the equations so far raised, the value of the network output can be generalised as follows:

(3) $$y_{i}=f\left(\sum_{j=1}^{n}w_{ij}x_{j}+\theta_{i}\right)$$

#### 2.5.1 Training algorithms

Since the objective of the network is to provide an output value as close to the observed value as possible, network learning is posed as a problem of minimising the output overall error defined by the function:

(4) $$E=\frac{1}{N}\sum_{n=1}^{N}e_{n}$$

where *N* is the number of training patterns and *e*_*n*_ is the error made by the network for pattern n defined as:

(5) $$e_{n}=\sum_{i=1}^{M}{\left(s_{i}-y_{i}\right)}^{2}$$

where *M* is the total number of output nodes, *y*_*i*_ the output value provided by the network at the *i*th output node and *s*_*i*_ the observed or target value at the *i*th output node. The function error depends exclusively on the parameters of the network (weights and biases). Depending on the value that these parameters take, more or less weight will be given to neurons and their connections with the successive layers. Therefore, by modifying weights of the input layer, the influence of each input variable on the output variable will be modified*.* These parameters can be grouped into a single vector of the corresponding dimension, which we will denote by *w*. In this sense, we can write the following expression in order to indicate that the value of the error made by the neural network will depend on this vector:

(6) $$e_{n}=f\left(w\right)$$

With this formalisation, the aim is to find the value w∗ for which a global minimum of the function f is obtained, turning the learning problem into an optimisation problem. If the function f meets sufficient derivability conditions, the first and second derivative of this function provides the gradient vector and the Hessian matrix respectively:

(7) $$\frac{\partial f}{\partial w_{i}},for\;i=1,\;\ldots ,N$$

(8) $$H_{ij}f\left(w\right)=\frac{\partial^{2}f}{\partial w_{i}\partial w_{j}},for\;i,j=1,\ldots ,N$$

From these equations, a point (network parameters) can be found for which the gradient vector is null and that represents a maximum or a minimum of the function f. On the other hand, for a minimum, some conditions of the Hessian matrix must be verified.

The most common neural network training methods are based on conditions of derivability of the error function. The gradient descent or backpropagation method [32] is one of the most popular and implemented for its ease and scope of application. This method propagates the error measured in the output layer to the hidden layers, adjusting the network parameters in the steepest descent direction, that is, the most negative of the gradients. It is, therefore, a first-order method since it only uses the gradient vector. However, although it is a simple algorithm, multiple iterations are necessary to verify that the direction chosen is the one that allows to reduce the error function and achieve the convergence as quickly as possible.

Second-order methods make use of the Hessian matrix to reduce the number of iterations necessary until reaching the convergence. However, the main disadvantage of these methods is the high computational cost involved in the calculation of the Hessian matrix. To overcome this limitation, alternative methods have emerged that make modifications in obtaining training directions or that propose approximations of the Hessian matrix to achieve faster and simpler convergence.

Here, some of the most implemented second-order training methods were used to test which of them produces better results and faster training for the case of study. Subsequently, these training algorithms are explained in more detail. To do this, we will denote as $w_{i}$ the network parameter vector in the iteration i, $f_{i}=f(w_{i}$*)* the error value in the iteration i and $g_{i}=\nabla f\left(w_{i}\right)$ the gradient value of the error function in the iteration i.

##### 2.5.1.1 Quasi-Newton methods

Newton’s method [33] makes use of the Hessian matrix to find the best directions of variation of the network parameters. This method allows generating the vector of parameters such as:

(9) $$w_{i+1}=w_{i}-H_{i}^{-1}g_{i}$$

If the Hessian is not defined positive, the succession of parameters might tend to a maximum instead of a minimum. To avoid this problem, the above equation may be modified as follows:

(10) $$w_{i+1}=w_{i}-\left(H_{i}^{-1}g_{i}\right)v$$

Thus, the method attempts to determine the training direction first and then, an appropriate training speed v. However, as explained above, the calculation of the Hessian matrix is complex and implies a high computational cost. The Quasi-Newton (QN) methods seek an approximation of the inverse of the Hessian matrix without the need to solve the second derivatives in each iteration, thus simplifying its calculation. If we denote this approximation for the iteration i as $G_{i}$*,* the Quasi-Newton method can be defined as follows:

(11) $$w_{i+1}=w_{i}-\left(G_{i}\;g_{i}\right)v$$

The most popular methods for the calculation of $G_{i}$ are the Davidon-Fletcher-Powell formula [34], [35] and the Broyden-Fletcher-Goldfard-Shanno formula [36], which will be used to train our network.

##### 2.5.1.2 Levenberg-Marquardt algorithm

Like the Quasi-Newton methods, the Levenberg-Marquart (LM) algorithm [37] was also developed to perform faster training without calculating the exact Hessian matrix. When the error function approaches a sum of squares as happen in feed-forward networks, then the Hessian matrix can be approximated as follows:

(12) $$H=J^{T}J$$

where J is the Jacobian matrix that contains the first derivatives of the error function concerning weights and biases. The gradient is obtained as follows:

(13) $$g=J^{T}e$$

where *e* is the network error vector. The LM method updates the parameters vector for each iteration by using the following equation:

(14) $$w_{i+1}=w_{i}-{\left[J_{i}^{T}J_{i}+\mu I\right]}^{-1}J_{i}^{T}e_{i}$$

where *μ* is the learning rate. When *μ* is null, the algorithm becomes Newton’s method (Eq. (9)). The LM algorithm seems to be the fastest method to train moderately sized feed-forward networks (up to several hundred parameters). Its main disadvantage is that it requires storing the Jacobian matrices that can be very large for certain data sets. This fact results in a large use of memory.

### 2.6 Conjugate Gradient

The Conjugated Gradient (CG) method seeks to speed up the convergence concerning the traditional CG method without calculating the Hessian matrix. The traditional gradient descent method defines as training direction the one that faster minimises the error function. However, this does not necessarily imply that the fastest convergence occurs. To that end, the CG method uses conjugate training directions that generally produce faster convergence directions [38]. The set of training directions is defined by the following equation:

(15) $$d_{i+1}=g_{i+1}+d_{i}\gamma_{i}$$

where γ is the conjugate parameter, for which different authors have proposed alternative methods of calculation [39], [40]. Finally, the parameters of the error function are obtained as follows:

(16) $$w_{i+1}=w_{i}+d_{i}v$$

In the next section, the three training algorithms are compared according to their convergence velocities in training and performances in testing.

## 3 Results and discussion

### 3.1 Algorithm analysis

Three MLPs were developed for testing each training algorithm and compared its performance by using MATLAB version R2017b as the computational platform. The optimal number of neurons in the hidden layer cannot be known in advance and must be obtained empirically. This is a key factor since it significantly influences the ability of the network to generalise from training data [41]. A small number of neurons could lead to poor training (under-fitting) while too many neurons can lead to overtraining (over-fitting) [42]. That is the reason why different MLP topologies were defined by varying the number of neurons in the hidden layer from 3 to 12. Each algorithm was tested with each configuration and a total number of 100 runs were performed per algorithm and configuration to find the winning model. After each iteration, the values of the weights were saved and the model was validated through performance metrics. The experimental data set was divided into 3 subsets: 60% of the data was used for network training with the selected algorithm, 20% to validate the model and warn of under-fitting and over-fitting problems, and the remaining 20% to evaluate the accuracy of the model. Finally, a maximum number of 100 epochs was established and the training was stopped after six consecutive increases in validation error and the best performance was taken from the epoch with the lowest validation error.

Table 2 shows the best results obtained for each training and configuration algorithm: the correlation coefficient (R) measures the degree of association between two variables, in this case, the power value observed and predicted by the network; the mean squared error (MSE) allows us to know the amount of error that exists between the two data sets of both variables; and CPU time measures the speed of convergence, which means the time needed by each algorithm to find a solution that meets the training stop criteria. The topologies that exhibited the best results for the QN, LM and CG algorithms were those that had 9, 8 and 11 neurons in the hidden layer, respectively.

**Table 2:** Performance results of the training algorithms in the testing period.

Neurons Hidden layer	QN			LM			CG		
	R	MSE (μW.cm^−2^)	Time (s)	R	MSE (μW. cm^−2^)	Time (s)	R	MSE (μW cm^−2^)	Time (s)
3	0.886	10.32	11.4	0.862	9.38	7.2	0.791	9.54	23.2
4	0.888	8.88	10.8	0.861	10.13	9.5	0.869	7.88	16.8
5	0.884	7.56	9.1	0.892	7.94	11.3	0.888	9.51	9.2
6	0.901	10.20	18.0	0.878	7.46	17.3	0.898	8.07	23.6
7	0.894	11.25	13.3	0.928	9.15	14.6	0.856	10.64	27.4
8	0.895	8.45	14.3	0.950	7.90	7.8	0.898	8.56	14.1
9	0.932	7.89	11.2	0.920	8.79	9.6	0.901	8.18	34.3
10	0.779	12.56	8.4	0.946	7.67	13.5	0.913	7.66	27.0
11	0.898	8.65	27.6	0.916	7.80	18.5	0.920	10.07	18.2
12	0.895	7.78	16.6	0.884	7.72	15.4	0.841	10.13	17.6

**Fig. 3:**
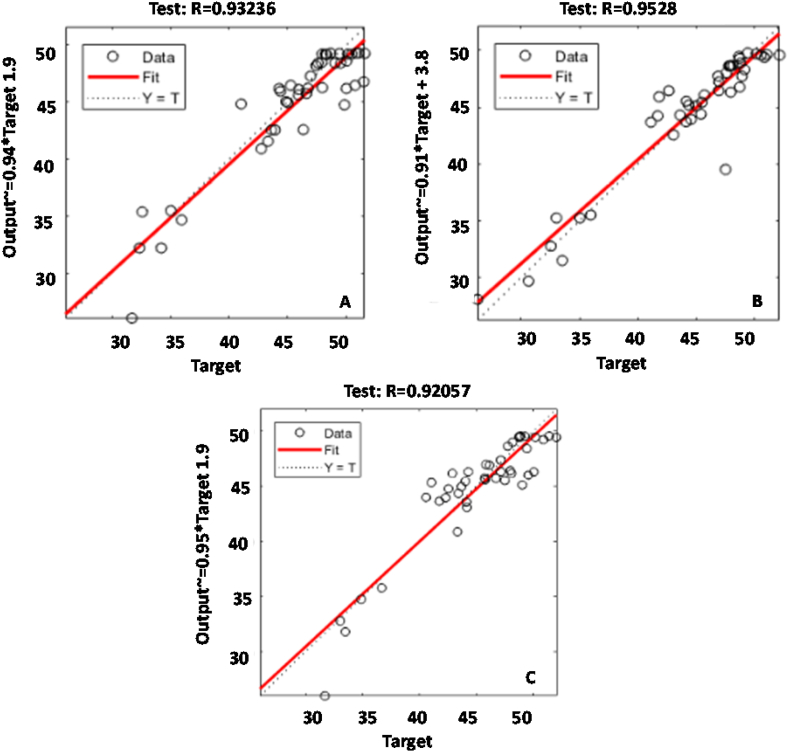
Regression plot of observed and predicted power in testing period: (A) QN, (B) LM, (B) CG.

Fig. 3 shows the scatter plot of the winning models of each algorithm. For each of them, the fit between the predicted model and the observed data was fairly precise, and therefore represented a strong linear correlation between both data sets. The LM algorithm was the one that obtained the highest accuracy in the adjustment (greater than 95%), with an MSE of 7.90 in the testing period and convergence time of 7.8 s. The QN algorithm obtained an R and MSE of 0.932 and 7.89, respectively, for a time of 11.2 s. On the other hand, the CG algorithm was the one that obtained the lowest R (0.92) with an MSE of 10.7 and convergence time of 18.2 s.

**Fig. 4:**
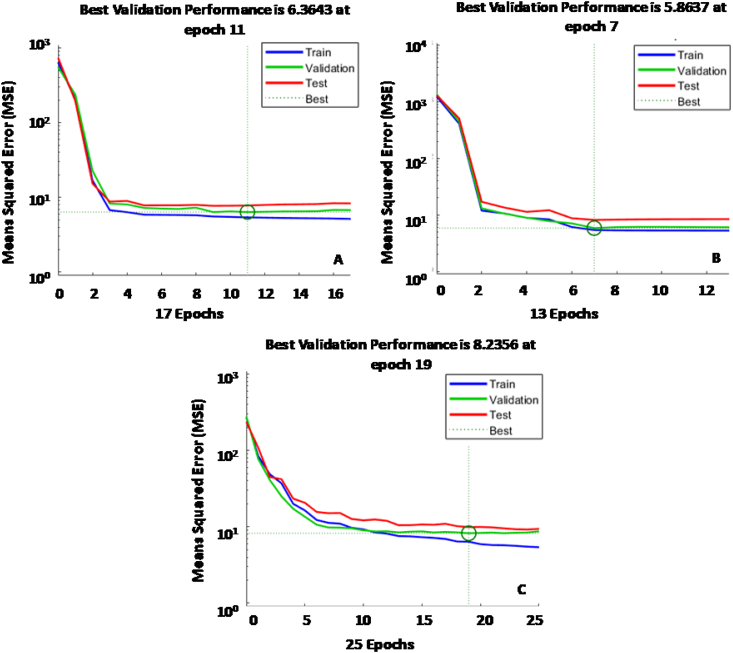
MSE for each winning model in validation period: (A) QN, (B) LM, (C) CG.

In general, the three models offered good fit accuracy and a small MSE, indicating little deviation between the observed value and the one predicted by the network. In fact, the LM, QN and CG algorithms made a total of 85%, 82%, and 89% predictions respectively, with a relative error lower than 10%. Moreover, the validation error for each algorithm was low and slightly higher than the training error, which rules out under-fitting and overfitting problems (see Fig. 4). In light of these results, it can be concluded that the LM algorithm was the one that best and fastest solved the power prediction problem in MFCs.

### 3.2 Analysis of the experimental and simulated results

**Fig. 5:**
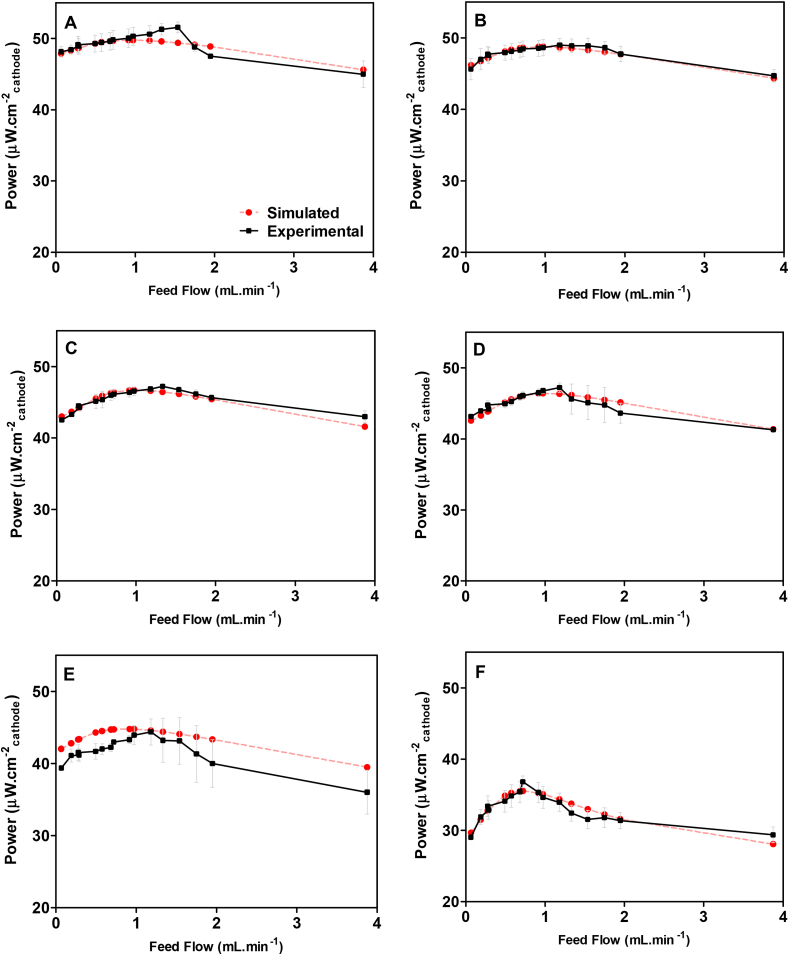
Comparison of the simulated and the mean experimental power performance of ceramic MFCs: A) Group 1, B) Group 2, C) Group 3, D) Group 4, E) Group 5, F) Group 6, previously described.

According to the previous discussion, the algorithm which allows us to predict the power performance of the MFCs more accurately is LM (R = 0.95%). Fig. 5 shows the experimental results obtained by feeding the 6-groups of different ceramic MFCs with neat human urine at 16 target feed flow rates. As can be observed, the experimental power output by the groups 1, 2, 3 and 4 is very similar, being group 1, which achieved the maximum value. This result might be related to the low bulk resistance of the ceramic membrane of this group (64 Ω). However, the performance of these four groups showed a similar trend, probably due to the porosities of the ceramic membranes being quite similar and the bulk resistances being relatively low. All of them reached the maximum power output at feeding flows ranged between 1.18 and 1.53 mL min^−1^, although MFCs belonging to group 1 were the only which kept increasing the power performance for flow rates higher than 1.33 mL min^−1^. After these values of flow rate, the power performance of these four groups decreased until reaching the initial power output or even lower. These results might be due to low flow rates of fuel supply are limiting and the fluidic shear rate is low, promoting the formation of thick and dense biofilms, giving rise to diffusion-limitation of the substrates. By contrast, if the flow rate-shear rate is higher, the thickness of the biofilm is reduced by the detachment of outer layers of cells, and the current produced increases due to higher supply rate of limiting nutrient and/or removal of diffusion-limiting outer layers of biofilm. For these reasons, an increase in the flow rate initially facilitates the development of a more efficient biofilm, which improve the power performance by the system [43]. However, if the flow rate is very high, the current produced might be reduced due to cell detachment of the inner core layer of cells [[44], [45], [46], [47]].

In the case of groups 5 and 6, both exhibit lower values of power output than the groups previously commented, probably because the bulk resistance of the ceramic membranes used in these cases are significantly higher. It is worth mentioning that the performance of MFCs working with the least porous membrane (16.8%) and the highest bulk resistance (497.2 Ω) was more sensitive to changes in the feed flow rate. For feeding flow rates ranging between 0.06 and 0.72 mL min^−1^ the power performance increased significantly, showing higher slope that in the rest of groups. However, once the flow rate was higher than 0.72 mL min^−1^, power output decreased dramatically, almost at a level lower to that achieved following the previous increase. These results might be related to the blockage of the pores, which could happen easily than in other membranes with higher porosities. In this case, the maximum power output was 28.6%, lower than that observed in group 1 and it was obtained at a feed flow of 0.72 mL min^−1^, less than the half of flow rate needed by the group 1 to reach the best performance.

## 4 Conclusions

The flow rate, and therefore, the hydraulic retention time and the shear stress significantly affect the power performance of MFCs. For this reason, this work aims at simulating the effect of the neat urine flow rate on the power performance of ceramic MFCs by using an ANN approach. To this end, a multilayer perceptron, one of the most commonly used ANN models worldwide, was designed. Three second-order algorithms, the QN, LM and CG were used to train our network. These methods represent an advantage in terms of computational cost regarding other second-order algorithms, such as the backpropagation method, since they use approximations of the Hessian matrix or modifications in the training directions that speed up the network learning. Each of these algorithms was tested for different network topologies until the best model was found. In general, the three training algorithms tested were able to accurately simulate the power prediction, showing a small MSE and convergence time, proving to be suitable algorithms for this case study. Among all of them, the LM was the one that presented the highest accuracy (R = 95%) and the fastest convergence (7.8 s). These results show that ANNs are useful and reliable tools for predicting energy harvesting from ceramic-MFCs under changeable flow rate conditions, which will facilitate the practical deployment of this technology.

## Credit author statement

**A. De Ramón-Fernández and MJ. Salar-García:** Conceptualisation, Data curation, Formal analysis, Investigation, Methodology, Visualisation, Writing - original draft, Writing - review & editing. **D. Ruiz Fernández:** Funding acquisition, Supervision, Writing - review & editing. **J. Greenman:** Supervision, Writing - review & editing **I. Ieropoulos:** Conceptualisation, Funding acquisition, Project administration, Resources, Supervision, Writing - original draft, Writing - review & editing.

## Declaration of competing interest

The authors declare that they have no known competing financial interests or personal relationships that could have appeared to influence the work reported in this paper.
